# Adaptive Template Update and Re-Detection Network Based on Tracking Confidence

**DOI:** 10.3390/s26103251

**Published:** 2026-05-20

**Authors:** Wanxin Wu, Yuxuan Ding, Kehua Miao

**Affiliations:** Department of Automation, Xiamen University, Xiamen 361102, China

**Keywords:** object tracking, Siamese network, template update

## Abstract

Siamese tracking is widely used in object tracking due to its efficient dual-branch symmetric structure, deep feature matching mechanism, and flexible template strategy. Existing mainstream Siamese tracking algorithms typically employ static template matching or linear combination-based template updating to localize the target in the next frame. However, these mechanisms often struggle to ensure template accuracy in complex environments involving changes in target appearance, scale, occlusion, and motion blur, thereby compromising robustness and stability. To address these issues, this paper proposes a confidence-guided adaptive template update with a re-detection (CATUR) network. CATUR constructs a tracking confidence assessment module that uses average peak-to-correlation energy (APCE) and a dynamic threshold mechanism to determine the current tracking state, providing a basis for template updates and target re-detection. It also designs an adaptive template update network that effectively combines the initial, historical, and current-frame templates, enhancing adaptation to target appearance variations. By integrating a global search module and a re-detection module, CATUR achieves precise target re-localization, rapid template updating, and tracking recovery. Extensive experiments and ablation studies on LaSOT and TrackingNet demonstrate that CATUR improves AUC, PNorm, and P by 4.0%, 4.0%, and 3.2%, respectively, significantly enhancing tracking accuracy and robustness in complex environments.

## 1. Introduction

In visual object tracking, the static template-matching mechanism constructed from the initial frame serves as the core tracking strategy for most tracking algorithms. Siamese tracking models, such as SiamFC [1], SiamRPN++ [2], SiamDW [3], and MFFSiam [4], are based on this mechanism and typically maintain high tracking accuracy and stability in short-term tracking tasks where the target’s appearance changes only slightly [5,6]. However, in real-world applications, target features undergo complex changes, including appearance deformation, partial occlusion, and background distraction. In tracking scenarios involving extended durations or targets whose appearance continuously changes, the static template matching mechanism, due to the absence of an update mechanism, suffers from a growing representation discrepancy between the fixed template and the dynamic target, which accumulates over time, ultimately leading to target drift or even tracking failure [7,8,9]. Evaluation results from long-term datasets such as LaSOT [10] also indicate that existing tracking algorithms still have room for improvement when dealing with extended tracking periods or complex changes.

To address the above issues, some tracking algorithms based on Siamese networks employ a dynamic template matching mechanism that extracts the target template from the current frame and updates it frame by frame. Starting from the initial stage of tracking, these methods typically fuse the current frame template with historical templates using linear weighting, thereby enhancing the model’s ability to adapt to changes in the target’s appearance to some extent [11,12]. However, as Zhang et al. pointed out in their paper UpdateNet [13], simple linear fusion strategies often lack the ability to assess template quality, making them susceptible to introducing noise and errors. This leads to template information degradation and reduced representational capacity, which in turn causes the model’s performance to degrade. Therefore, ensuring the representational capacity of the template while achieving stable template updates has become key to improving the performance of tracking algorithms.

This paper presents several key contributions:

1. We developed an adaptive template fusion network capable of effectively integrating information from initial, historical, and current templates, and supporting the triggering of re-detection and template update mechanisms in target-loss scenarios.
2. We effectively integrate Average Peak-to-Correlation Energy (APCE) into the proposed tracking framework and design a tracking confidence assessment module, which quantitatively evaluates the contrast between the target peak and the background in response maps and comprehensively analyzes the overall distribution characteristics of the response maps.
3. Experiments on the public datasets LaSOT [10] and TrackingNet [8] show that integrating the proposed CATUR network into a lightweight offline tracker achieves AUC scores of 54.7% and 74.9%, respectively. Ablation studies further validate the effectiveness and the practical value of the tracking confidence assessment module, confidence-guided adaptive template fusion and target-loss re-detection strategy in complex tracking scenarios.

## 2. Related Work

### 2.1. Template Update Mechanism

Depending on whether the target model or template is updated online during the inference stage, object tracking methods can be categorized into two types: offline tracking and online-updating tracking.

Offline trackers are typically trained on large-scale datasets to obtain a fixed model. This model remains unchanged during the tracking process and uses the model parameters learned during training to efficiently localize targets [5,7,14,15,16]. Offline training tracking models, represented by deep Siamese networks, acquire excellent target representation capabilities during training, thereby enabling real-time tracking during the inference stage through a fixed template matching mechanism [6,17].

SiamFC [1] was the first to adopt a fully convolutional Siamese network architecture in the field of object tracking. By calculating the similarity between the template and the search region through cross-correlation, it achieved an excellent balance between accuracy and speed. SiamRPN [18] further introduced a region proposal network, transforming object tracking into a one-shot detection problem and effectively improving localization accuracy. Building on this, SiamRPN++ [2] improved the application of deep backbone networks within the Siamese tracking framework and further enhanced tracking performance through layer-wise and depth-wise aggregations. Subsequently, C-RPN [19] introduced a cascaded region proposal network that progressively refines candidate bounding boxes, enhancing robustness across multiple scales. SiamMask [20] extended the Siamese architecture by adding a segmentation branch, enabling simultaneous visual tracking and semi-supervised video object segmentation. SiamBAN [21] and SiamCAR [22] further developed anchor-free and box-adaptive bounding box prediction frameworks, respectively.

TransT [23], proposed by Chen et al., introduced the Transformer architecture, replacing traditional correlation operations with self-attention and cross-attention to more fully model the relationship between the template and the search region. Although offline tracking methods that do not update the template offer significant advantages in terms of computational efficiency and stability, their adaptability is limited when the target appearance undergoes significant changes.

In contrast, online-updating tracking methods continuously update the model or target template based on the latest target information during the tracking process, enabling the tracking model to respond in real time to changes in the target’s appearance, scale, and background, and demonstrating greater robustness in long-term tracking scenarios.

Early works such as DaSiamRPN [24] adopted a linear update strategy that performed weighted fusion of the historical template and the current-frame template using fixed weights. While this approach featured a simple structure and high computational efficiency, it was prone to template drift under conditions such as occlusion or target deformation.

To address this issue, researchers have subsequently proposed various nonlinear fusion architectures based on deep neural networks. UpdateNet [13] utilized a convolutional neural network to learn a template fusion function, enabling end-to-end nonlinear updates of the historical template and the current template. Building on this, Fu et al. proposed STMTrack [25], which incorporated multi-frame historical information by constructing a spatiotemporal template memory pool and dynamically selecting the optimal template from it using an attention mechanism. Furthermore, Dai et al. proposed Long-Term Tracking with Meta-Updater (LTMU) [9], which improved the template management strategy by introducing an LSTM-based [26] policy module to adaptively select between long-term and short-term template sources.

Although the aforementioned methods enhanced the tracker’s adaptability to long-term appearance variations and occlusion scenarios, most models suffer from complex architectures and high computational costs, necessitating further exploration in terms of structural simplification and update strategies.

Building on the above, this paper proposes a confidence-guided adaptive template fusion method, which effectively balances computational cost and template update quality, enhances the model’s ability to model target appearance features, and improves the tracker’s robustness in complex scenarios to a certain extent.

Common methods for updating templates are shown in Table 1.

The template update variables are defined as follows. $T_{t}$ represents the template updated from the *t*-th frame; $T_{c}$ is the template extracted from the current frame; $T^{i}$ is the *i*-th template in the historical template pool; α is the linear fusion coefficient used to control the weight ratio between the current template and the historical template; f(·) is the nonlinear fusion function, typically implemented by a neural network; and $w_{i}$ is the attention weight, calculated as follows:

(1) $$w_{i}=\frac{\exp\;\left(\phi (T_{c},T^{i})\right)}{\sum_{j=1}^{N}\exp\;\left(\phi (T_{c},T^{j})\right)}$$

Here, ϕ(·,·) represents the similarity function between templates, such as the dot-product operation used in attention mechanisms.

### 2.2. Tracking Confidence Assessment

Frequent or indiscriminate updates to the template may introduce erroneous information and reduce the tracking stability [13]. Therefore, a reliable method for confidence assessment is particularly important.

Currently, most Siamese-network-based tracking models adopt a strategy that directly selects the point with the highest confidence score in the classification-branch response map as the target center [5,27]. This strategy reflects only local matching confidence and struggles to accurately assess the overall tracking status.

In correlation-filter-based tracking algorithms, a response map is typically generated to quantify the similarity between the filter kernel and the image to be tracked, with the response value at each location representing the likelihood that it is the target [28,29,30]. This method enables the distinction between target and background information during the modeling process, allowing for more precise extraction of target features.

Blome et al. proposed a method for visual object tracking using adaptive correlation filters, named MOSSE [30], which was the first to apply correlation filtering to single-object tracking. It also introduced a strategy for dynamically updating fusion weights based on confidence, using the Peak-to-Sidelobe Ratio (PSR) to quantify the reliability of tracking results.

The MOSSE method introduces the maximum response value F_max_ and PSR to evaluate the confidence of tracking results. The maximum response value is simply the maximum value directly taken from the response map, making it a straightforward and efficient approach. And the PSR measures the intensity difference between the peak and the sidelobe regions in the response diagram. Compared to other confidence metrics, it offers better resistance to interference. The formula is defined as follows:

(2) $$\mathrm{PSR}=\frac{F_{\max}-\mu_{\mathrm{side}}}{\sigma_{\mathrm{side}}}$$

Here, $F_{\max}$ denotes the maximum value of the response map, while $\mu_{\mathrm{side}}$ and $\sigma_{\mathrm{side}}$ represent the mean and standard deviation of the sidelobe region, respectively. The sidelobe region refers to the portion of the response map remaining after excluding the highest peak and its surrounding neighborhood of a certain size. The size of the excluded region can be adjusted according to the application scenario and the resolution of the response map, and it is typically set to a 3×3 window.

In correlation-filter-based tracking methods, other confidence metrics have also been shown to effectively improve tracking stability and parameter tuning accuracy. Wang et al. [31] proposed the Average Peak-to-Correlation Energy (APCE), a metric that quantifies the peak saliency and overall energy distribution of the correlation-filter response map to assess the confidence of tracking results.

Inspired by this idea, this paper draws on the concept of confidence assessment from traditional correlation-filter-based object tracking methods, introduces a similar confidence quantification method based on the Siamese network to evaluate the tracking state using the response map, and designs corresponding template update schemes based on different tracking states.

### 2.3. Backbone Network

**Figure 1:**
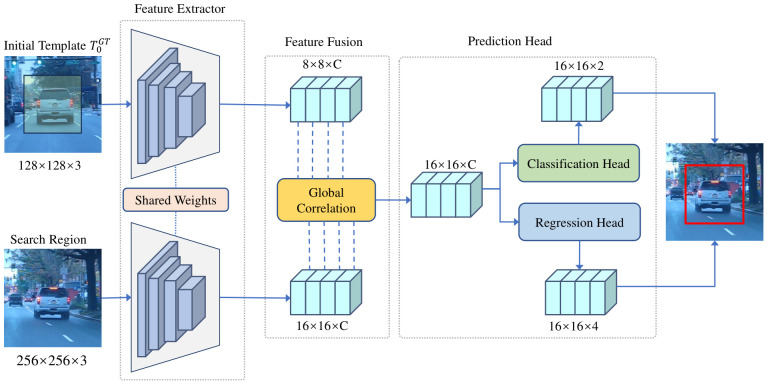
Architecture of the baseline SiamGCN. The template and search regions are processed by a shared feature extractor, fused by a global correlation module, and then sent to classification and regression branches.

To evaluate our proposed adaptive template update and re-detection modules, we adopt our previously published SiamGCN [32] as the baseline model, as shown in Figure 1. SiamGCN is a lightweight Siamese tracker with a MobileNetV3 [33] backbone and a global correlation module.

## 3. Proposed Method

**Figure 2:**
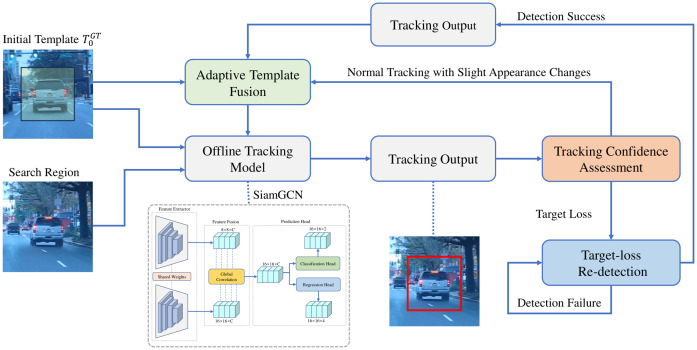
Overall framework of the confidence-guided template update network. The model primarily consists of four components: an offline tracking network, adaptive template fusion, tracking confidence assessment, and target-loss re-detection. Among these, the offline tracking network can be flexibly instantiated using any tracking network similar to SiamGCN.

The core idea of the proposed model is to build upon the offline Siamese tracking model by introducing tracking confidence to determine the current tracking state, and to dynamically adjust the template or tracking strategy accordingly. The overall framwork is in Figure 2.

During the model initialization phase, the target region in manually annotated first frame is used as the initial template. This template is manually annotated and typically exhibits extremely high reliability. Therefore, the initial template serves as a critical reference during subsequent template updating process. In the tracking process for each subsequent frame, the search region is input into the offline tracking model described earlier to obtain the tracking result for the current frame.

After obtaining the tracking results, the template update network utilizes a tracking confidence assessment module to evaluate the current tracking state. This module quantifies the peak saliency in the response map based on APCE metric, thereby determining whether the current template remains suitable for target matching in subsequent frames.

Our template update network categorizes the tracking state into three cases: normal tracking, drastic appearance variation, and target loss. If the confidence level of the tracking result is high, indicating only minor changes in the target’s appearance, the current state is classified as normal tracking. In this case, the adaptive template fusion network is used to update the template, ensuring that it adapts to changes in the target’s shape, scene illumination, and viewpoint shifts.

If the confidence level of the tracking result is low, the current state is classified as drastic target appearance variation. In this case, the model may still be tracking the target, but the reliability of the tracking result is insufficient; therefore, no template update is performed, and the template information in the adaptive template fusion network remains as the result of the last template update.

When tracking fails due to severe occlusion, rapid motion, or background interference, the proposed model employs global search and target detection to handle this situation. Once the current state is determined as target loss, that is, when the tracking confidence falls below the predefined threshold, the model triggers the target-loss re-detection mechanism. It then conducts a global search to re-localize the target and update the template, thereby restoring normal tracking.

### 3.1. Tracking Confidence Assessment

Without a template update mechanism, offline Siamese-network-based tracking models are inevitably affected by target deformation. While introducing template updates allows the model to adapt to changes in the target and background to some extent, indiscriminate template updating may lead to error accumulation and drift [30,34], which in turn leads to a decline in template accuracy.

Therefore, a reliable metric is needed to ensure that the model performs template updates only when tracking result is highly reliable. This approach allows the model to adapt to changes in the target and background while preventing low-confidence tracking results from introducing erroneous information. This paper adopts APCE as a quantitative measure of tracking result confidence, and its formulation is given as follows:

(3) $$\mathrm{APCE}=\frac{{\left(R_{\max}-R_{\min}\right)}^{2}}{\frac{1}{N}\sum_{i,j}{\left(R_{i,j}-R_{\min}\right)}^{2}}$$

Here, *R* represents the response map produced by the classification branch of the offline tracker for the current frame, while $R_{\max}$, $R_{\min}$, and $R_{i,j}$ denote its maximum value, minimum value, and the value at each corresponding position, respectively.

From Equation (3), it can be seen that APCE focuses on characterizing the difference between the maximum response and the other responses in the response map, using the prominence of the peak to reflect the confidence level of the tracking result.

**Figure 3:**
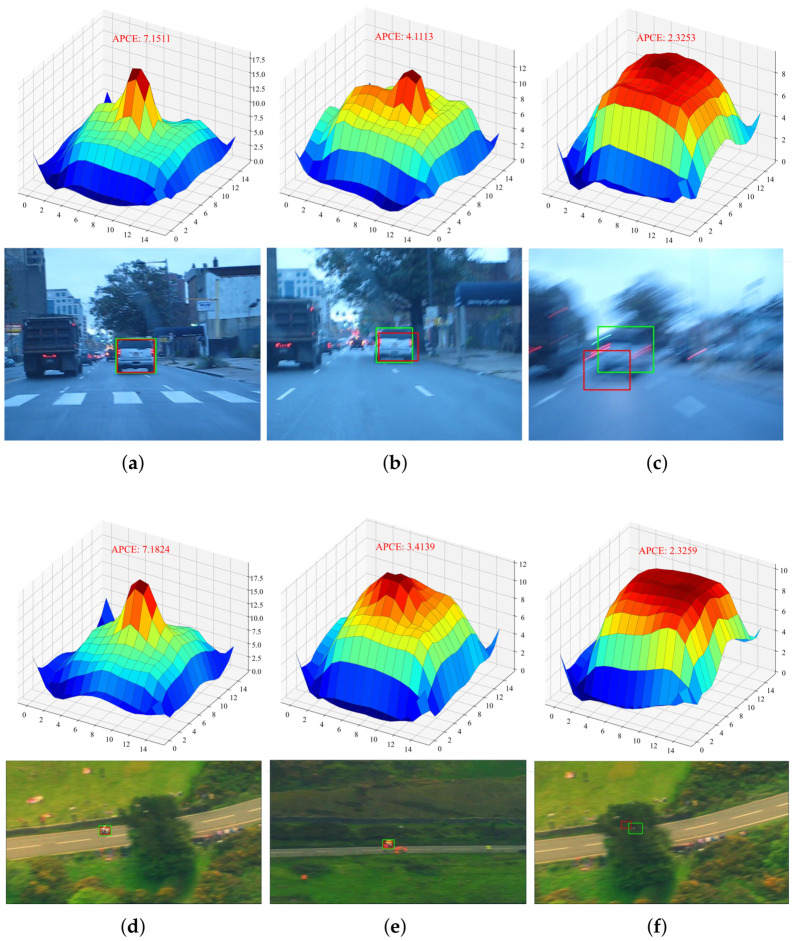
Visualization of peak response maps and APCE metrics under different tracking states. (**a**,**d**) denote normal tracking cases, (**b**,**e**) denote cases with slight target appearance changes, and (**c**,**f**) denote cases with drastic target appearance changes. Green bounding boxes indicate ground truth, and red bounding boxes indicate tracking results of the offline model.

The APCE values and corresponding response maps corresponding to different tracking states for the two video sequences are shown in Figure 3.

The two figures demonstrate that under normal tracking conditions, the response map exhibits a distinct sharp peak accompanied by a high APCE value. When the target is partially occluded or blurred, the response map displays multiple local peaks, and the APCE value decreases, indicating a reduced confidence in the tracking result. When the target is completely occluded, the response map lacks a prominent peak, the overall response is relatively uniform, and the APCE value is significantly lower. In contrast to the strategy proposed in this paper, most offline tracking models use the response map merely to map the point corresponding to its peak back to the original image as the target center. This single strategy approach cannot fully reflect the actual tracking state. Therefore, using APCE as a quantitative metric to evaluate tracking confidence is both feasible and necessary.

However, in actual testing, the distribution of APCE values across different video sequences varies significantly due to factors such as viewing angle, target size, and background complexity. For example, in the VOT2019 [35] dataset, the APCE values in the “Rabbit” sequence are primarily distributed between 2 and 7, while those in the “Soccer” sequence are primarily distributed between 3 and 10. Therefore, using a fixed APCE threshold to determine the tracking condition of the target across different sequences has certain limitations.

To this end, this paper proposes a dynamic threshold calculation method that allows the confidence boundaries to adapt to the APCE distribution of each sequence, rather than relying on a fixed value shared by all sequences. This method constructs a reference value using a weighted combination of the maximum APCE value over recent frames and the historical maximum APCE value, thereby dynamically determining the threshold for determining the tracking state. To ensure the system stability during the initialization phase and avoid directly using the historical maximum value, a pre-selected empirical value is adopted as the initial reference value, i.e.,

(4) $${\mathrm{APCE}}_{\mathrm{ref}}={\mathrm{APCE}}_{\exp}$$

This empirical value is obtained from offline experiments and provides a good representation of the target’s response map characteristics under ideal conditions.

In the subsequent tracking phase, a sliding window with a fixed size of *N* frames is defined, and the maximum APCE value within that window is calculated as:

(5) $${\mathrm{APCE}}_{\mathrm{recent}}=\max\left\{\mathrm{APCE}\left(t-N+1\right),\ldots ,\mathrm{APCE}\left(t\right)\right\}$$

Meanwhile, once initialization is complete, the historical maximum APCE value is updated as:

(6) $${\mathrm{APCE}}_{\mathrm{hist}}=\max\left\{{\mathrm{APCE}}_{\mathrm{hist}},{\mathrm{APCE}}_{\mathrm{recent}}\right\}$$

Then, the current APCE reference value is calculated by combining historical and recent information:

(7) $${\mathrm{APCE}}_{\mathrm{ref}}=\lambda \cdot {\mathrm{APCE}}_{\mathrm{hist}}+\left(1-\lambda \right)\cdot {\mathrm{APCE}}_{\mathrm{recent}},\;\lambda \in \left[0,1\right]$$

Here, the weighting factor λ is used to balance the influence of historical and recent APCE information on the reference value. The historical maximum helps preserve a stable confidence scale, while the recent maximum allows the threshold to respond to short-term changes in the current sequence. Therefore, the weighted formulation prevents the threshold from being dominated only by either long-term history or short-term fluctuations.

Finally, by setting the scaling parameters $\gamma_{1}$ and $\gamma_{2}$ (where $\gamma_{1}>\gamma_{2}$), the dynamic thresholds are constructed as:

(8) $$\alpha =\gamma_{1}\cdot {\mathrm{APCE}}_{\mathrm{ref}},\;\beta =\gamma_{2}\cdot {\mathrm{APCE}}_{\mathrm{ref}}$$

The two scaling parameters generate an upper threshold and a lower threshold, allowing the tracker to distinguish normal tracking, uncertain tracking, and target-loss states.

The tracking state is then determined according to the relationship between the current frame’s APCE(t) and the dynamic thresholds α and β:

1. When APCE(t)≥α, there is a distinct peak in the response map, the tracker is determined to be in a normal tracking state, and a template update can be performed;
2. When β≤APCE(t)<α, the response map may contain multiple local peaks, indicating that the tracker is in a state of drastic target appearance variation; in this case, the template information in the adaptive template fusion network will remain unchanged from the last template update;
3. When APCE(t)<β, the peak saliency in the response map significantly weakens, indicating that the current tracking state is target loss, which requires the re-detection mechanism to be triggered to re-localize the target.

Table 2 lists the hyperparameter settings involved. These parameters were set empirically based on the distribution of APCE in the video sequences used in the experiment.

Through the above process, the tracking model dynamically utilizes the tracking confidence of both the current frame and historical frames to provide a reliable basis for subsequent adaptive template fusion.

### 3.2. Adaptive Template Fusion

#### 3.2.1. Network Architecture

Traditional template update methods typically employ a weighted averaging strategy, in which the new template sample in each frame is linearly fused with the accumulated template from the previous frame. The update equation is generally formulated as follows:

(9) $${\tilde{T}}_{i}=\left(1-\alpha \right){\tilde{T}}_{i-1}+\alpha T_{i}$$

**Figure 4:**
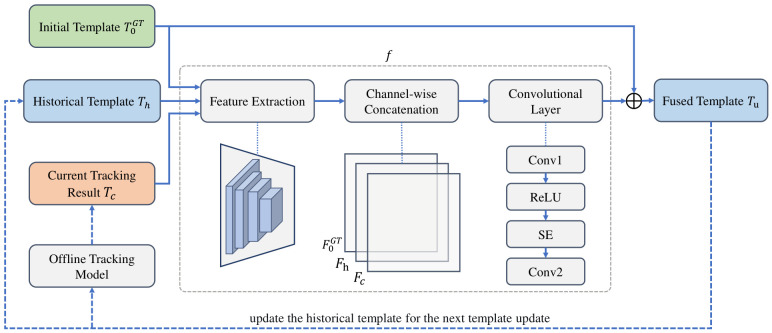
Adaptive template fusion network framework.

Here, $T_{i}$ represents the new template extracted from the tracking result of the current frame, ${\tilde{T}}_{i}$ is the accumulated template, and ${\tilde{T}}_{i-1}$ is the historical accumulated template from the previous frame. The update rate α is typically set to a small fixed value (e.g., 0.01). This strategy can, to some extent, adapt to smooth changes in the target appearance across consecutive frames. However, when the target appearance changes significantly or partial occlusion occurs, this linear fixed-rate update approach is prone to missing critical appearance information or introducing background noise. To address these shortcomings, this paper proposes a lightweight adaptive update network, whose structure is shown in Figure 4. The update network can be formulated as follows:

(10) $$T_{u}=f\left(T_{0}^{GT},T_{h},T_{c}\right)$$

Here, $T_{0}^{GT}$ is the initial template annotated in the first frame, $T_{h}$ is the historical accumulated template from the previous frame, $T_{c}$ is the target template extracted from the tracking result of the current frame, and $T_{u}$ is the updated template for the current frame, which serves as the cumulative historical template for the next update. The update function *f* is a convolutional neural network used for template fusion.

The adaptive template fusion network is designed as a lightweight fusion module to model the complementary differences among $T_{0}^{GT}$, $T_{h}$, and $T_{c}$, and to support adaptive template updating. The network first uses the feature extraction backbone in the offline tracking model to extract features from these three inputs, yielding three sets of features $T_{0}^{GT}$, $T_{h}$, and $T_{c}$, each of size H×W×C. When SiamGCN is used as the offline tracking model, H=W=8 and C=96. Subsequently, the three sets of features are concatenated along the channel dimension, resulting in a feature representation of size H×W×3C, and the concatenated features are processed sequentially through a series of convolutional layers. The first convolutional layer (Conv1) uses a 1×1×3C×96 convolution kernel to reduce the dimensionality of the features from H×W×3C to H×W×96. A ReLU activation function and an SE module [36] are then applied to recalibrate the channel-wise information. The second convolutional layer (Conv2) restores the original number of channels, with a kernel size of 1×1×96×C, producing an output feature of size H×W×C.

Feature concatenation preserves the feature information of each template prior to fusion. The subsequent convolutional layer learns cross-template channel interactions at a relatively low computational cost while adjusting the channel dimension. The SE module recalibrates channel responses, emphasizing high-information-content template features while suppressing noisy features. Consequently, this module provides an adaptive method for fusing multi-template information within a simple architecture.

Feature concatenation preserves the individual information of each template prior to fusion, the subsequent convolutional layers can then learn cross-template channel interactions at a lower computational cost, while the SE module further recalibrates channel responses to emphasize high-information-content template features and suppress noise features. Consequently, this module provides an adaptive method for fusing multi-template information within a compact architecture.

Given the high reliability of the manually annotated initial target position, this network introduces a residual connection [37], which integrates a skip connection from the initial template $T_{0}^{GT}$ into the output. This ensures that during updates, the network can integrate the historical appearance information of the tracked target while using the most reliable sample as the basis for updates.

The adaptive template fusion network not only effectively integrates target appearance information across different time steps but also employs a channel attention mechanism to achieve adaptive feature weighting, enhancing the robustness and discriminative power of template updating.

#### 3.2.2. Construction of the Loss Function

The training objective of the adaptive template fusion network is to predict the target template for the next frame. The predicted template should match the template extracted from the ground-truth target location in the next frame. Accordingly, this paper defines the loss function as the Euclidean distance between the two, calculated as shown in Equation (11):

(11) $$L={∥T_{u}-T_{u}^{GT}∥}_{2}={∥f\left(T_{0}^{GT},T_{h},T_{c}\right)-T_{u}^{GT}∥}_{2}$$

where $T_{u}$ represents the output of the template fusion network, and $T_{u}^{GT}$ denotes the ground-truth template extracted from the ground-truth target location in the corresponding frame. The template fusion network minimizes the Euclidean distance between the predicted template and the ground-truth template via a loss function. It can be trained as an independent module before being integrated into the tracking model.

During network training, the target template $T_{0}^{GT}$ for the initial frame and the template $T_{u}^{GT}$ for the target frame can be obtained directly by extracting features from the ground-truth target locations in the corresponding frames. However, due to the limited accuracy of the tracking model in estimating the position in the current frame, the template $T_{c}$ for the current frame cannot be obtained directly from the ground-truth target locations. Using the true position directly would tend to assume only negligible variation in $T_{c}$, thereby preventing the fusion network from learning effective update parameters. To address this issue, this paper constructs $T_{c}$ samples by introducing a certain amount of localization error during training to simulate the localization deviations that may occur during actual tracking. Additionally, this paper uses the accumulated template $T_{h}$ for feature extraction to simulate the accumulation of errors that may be encountered during tracking.

### 3.3. Target-Loss Re-Detection

**Figure 5:**
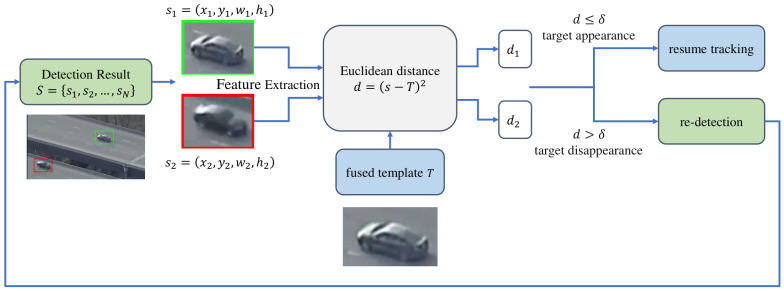
Target-loss re-detection process. Objects correctly detected are shown within green boxes, while objects within red boxes are distractor detections that share the same category as the template and have a similarity score exceeding the threshold.

In practical tracking scenarios, temporary loss of the target is a common issue. Currently, most offline tracking methods rely on local neighborhood information for re-localization, a strategy with a very limited search range. When the target reappears in a distant region of the frame, the model may fail to capture it quickly or even get stuck in a local loop. To address this, this paper introduces a global re-detection mechanism for target-loss scenarios, building upon the previously described tracking state judgment and adaptive template fusion. This mechanism utilizes an efficient object detection algorithm to search for candidate regions matching the target’s class across the entire video frame, thereby providing candidate regions for subsequent template-based verification and enabling rapid re-localization of the lost target. The arget-loss re-detection process is shown in Figure 5.

When the tracking confidence module determines that the current state is target loss, this paper employs an external object detector NanoDet-plus [38] to detect all objects of the same class as the target in the entire image. It should be clarified that NanoDet-plus is used to provide same-category candidate regions, and the final target is determined by comparing the normalized feature of each detected candidate with the fused template. It then uses the Euclidean distance between the detection results and the fused template as a similarity metric to determine whether the target has reappeared.

In the re-detection mechanism, the initial template is first identified using the object detection network NanoDet-plus, and the template’s category information is recorded. Next, the system detects objects of the same category as the template in the current search frame, yielding a series of detection boxes $S=\{s_{1},s_{2},\ldots ,s_{n}\}$, where each detection box $s_{i}$ contains the position and scale information $\left(x_{i},y_{i},w_{i},h_{i}\right)$ of a same-category object in the image. Subsequently, each detection box is processed through feature extraction and normalization via the offline tracking network. The Euclidean distance is then calculated between the fused template *T* and each detection result. If the Euclidean distance between a detection result and the template is less than the set threshold δ(δ=0.7), that detection result is considered the tracked target. If multiple detection results have Euclidean distances from the template that are all less than the threshold δ, the one with the smallest Euclidean distance is selected. At this point, the model outputs the target bounding box and exits the re-detection mechanism, after which the result is fed into the adaptive template fusion module to resume the tracking process in the next frame. If the Euclidean distances between all detection results and the template are greater than the threshold δ, it indicates that the tracking target has not yet been detected; the model will remain in the target-loss state and perform re-detection in the next frame.

## 4. Experiments

### 4.1. Implementation Details

The hardware and software configurations used in this study are shown in Table 3. All experiments were conducted in the above environment, which not only meets the computational demands of deep learning but also ensures the reliability and consistency of the results.

#### 4.1.1. Training of the Adaptive Template Fusion Network

The adaptive template fusion network can be regarded as a predictive model whose task is to predict the ground-truth template of the next frame. During network training, this paper constructs a set of templates from the same video sequence as input, including the initial template $T_{0}^{GT}$, the historical accumulated template from the previous frame $T_{h}$, the template extracted from the current tracking result $T_{c}$, and the ground-truth template of the next time step $T_{u}^{GT}$. Among these, $T_{h}$ and $T_{c}$ are generated during the actual tracking process, while $T_{0}^{GT}$ and $T_{u}^{GT}$ are derived from the ground truth annotations in the dataset. The training data are selected from GOT-10k [39], from which 20 categories were randomly sampled. Twenty training sequences were selected from each category to construct a category-balanced training subset.

The training process was divided into an initial training phase and a subsequent training phase.

In the initial training phase, templates were extracted from the tracking results generated by an offline tracking model without a template update mechanism for each frame, and these templates were used as the initial inputs. The network weights for this phase are initialized from scratch, the learning rate decayed logarithmically from $10^{-6}$ to $10^{-7}$ per epoch, the batch size is set to 64, and the model is trained for a total of 50 epochs.

In the subsequent training phase, templates are extracted using the output from the adaptive template fusion network of the previous training phase. The network weights in this phase are initialized using the best model from the previous phase, and the learning rate decayed logarithmically from $10^{-7}$ to $10^{-8}$ per epoch. The optimizer was stochastic gradient descent (SGD), with a weight decay parameter of 0.0005 and a momentum coefficient of 0.9.

A total of three training cycles were conducted: one initial training cycle that extracted templates from the tracking results of SiamGCN, and two training cycles that extracted templates from the output of the adaptive template fusion network of the previous stage.

Additionally, when applying the template update network described in this paper to offline tracking models, the corresponding feature extraction backbone of each model were used to ensure the consistency of the overall system and the comparability of evaluation results, enabling the adaptive template update network to be integrated as a standalone module into existing offline tracking models.

#### 4.1.2. Training of the NanoDet-Plus Detector

This paper adopts NanoDet-plus as the object detection model, which is primarily used for target-loss re-detection in tracking tasks; therefore, no excessive training tricks were used during training. The detection model was trained on the COCO dataset [40] using a series of data augmentation strategies, including random scaling, random stretching, random flipping, translation, and adjustments to brightness and saturation. The optimizer used was AdamW [41], with an initial learning rate set to 0.001 and a weight decay coefficient of 0.05. During the initial training phase, a warm-up learning rate strategy was employed to gradually increase the learning rate from 0.0001 to the initial value. The total training duration was 300 epochs, during which the learning rate was periodically adjusted using a cosine annealing strategy [42], the $T_{max}$ is 300 and the minimum learning rate is $5\times 10^{-5}$.

After training, the model achieved a detection performance of mAP(0.5:0.95)=26.9 on the COCO dataset, providing a reliable basis for target-loss re-detection.

### 4.2. Results and Comparison

**Figure 6:**
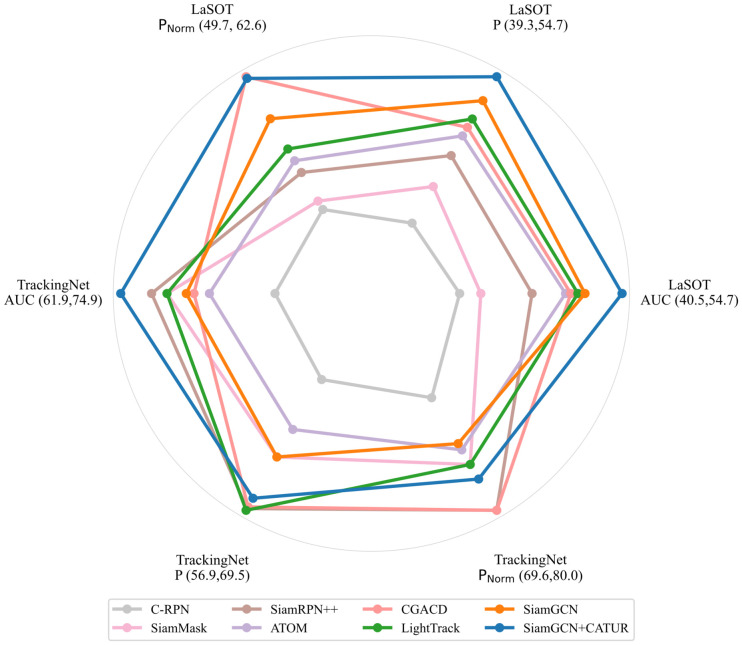
Detailed comparisons on LaSOT and TrackingNet. To improve visual clarity, Figure 6 omits the first two models from Table 4 and normalizes all evaluation metrics, the normalization ranges are indicated in the figure.

In this paper, we conduct a performance comparison between the proposed tracking algorithm and current state-of-the-art (SOTA) models, using two mainstream datasets: LaSOT [10] and TrackingNet [8]. These datasets cover a variety of challenging scenarios in the field of object tracking, such as fast motion, occlusion, long-term tracking, and complex background environments. The experimental results of our model and various SOTA models on the LaSOT and TrackingNet datasets are shown in Table 4 and Figure 6. The evaluation metrics include Area Under the Curve (AUC), Normalized Precision (PNorm), and Precision (P).

SiamGCN achieved AUC, PNorm, and P of 52.6%, 60.1%, and 53.0%, respectively. After integrating the confidence-guided adaptive template update and re-detection network (CATUR) proposed in this paper, SiamGCN + CATUR improved its AUC to 54.7%, a 4.0% increase over the SiamGCN, PNorm and P increased to 62.5% and 54.7%, respectively, representing improvements of 4.0% and 3.2% over the baseline. The experimental results validate the effectiveness of our method in enhancing tracker performance.

Compared to the LaSOT dataset, TrackingNet features a greater variety of object categories and scene variations, but the range of object scale variations is relatively small. Consequently, the overall performance of the tracking models has improved. SiamGCN + CATUR achieved AUC, PNorm, and P values of 74.9%, 78.5%, and 68.8%, respectively, representing improvements of 4.8%, 2.2%, and 3.6% over SiamGCN. The experimental results demonstrate that the CATUR network effectively enhances tracking robustness on TrackingNet, a long-term tracking dataset covering diverse scenarios.

Overall, Figure 6 shows that our model achieves strong overall performance on both datasets. On LaSOT, our model ranks second in PNorm and outperforms other SOTA models in both AUC and P. On the TrackingNet dataset, our model’s overall performance is slightly inferior to that on LaSOT, but it still remains at a leading level in the AUC metric. Furthermore, some methods that performed well on TrackingNet, such as SiamRPN++, demonstrated significantly lower performance on the LaSOT dataset compared to SiamGCN + CATUR. The performance gains on LaSOT and TrackingNet are moderate, and the consistent improvements demonstrate that CATUR can enhance the baseline tracker in complex long-term tracking scenarios, particularly in situations involving temporary target loss, occlusion, distractor interference, and drastic appearance changes. In these scenarios, response maps may contain multiple local peaks or unreliable target responses, and simple template updates may introduce noise. In contrast, CATUR can suspend unreliable template updates through the tracking confidence mechanism and trigger global re-detection when target loss is detected.

The additional computational cost introduced by CATUR primarily stems from three components: APCE-based confidence evaluation, adaptive template fusion, and re-detection using NanoDet-plus. Among these, APCE computations are performed directly on the response map and mainly involve simple maximum, minimum, and summation operations, so the computational overhead is limited. For the adaptive template fusion network, when SiamGCN is used as the offline tracker, the feature size is H=W=8 and C=96. Theoretically, the two main convolutional operations in the fusion module introduce a total of approximately 2.36M MACs, excluding lightweight activation functions and SE operations. Compared with APCE computations and the adaptive fusion network, the re-detection mechanism using NanoDet-plus may introduce greater additional overhead when triggered. However, NanoDet-plus itself is a lightweight detector and is activated only when the tracking confidence module determines that the target is lost, rather than being applied to every frame. Therefore, the additional runtime overhead introduced by the re-detection component is closely related to the frequency of target-loss events.

### 4.3. Visualization Analysis

**Figure 7:**
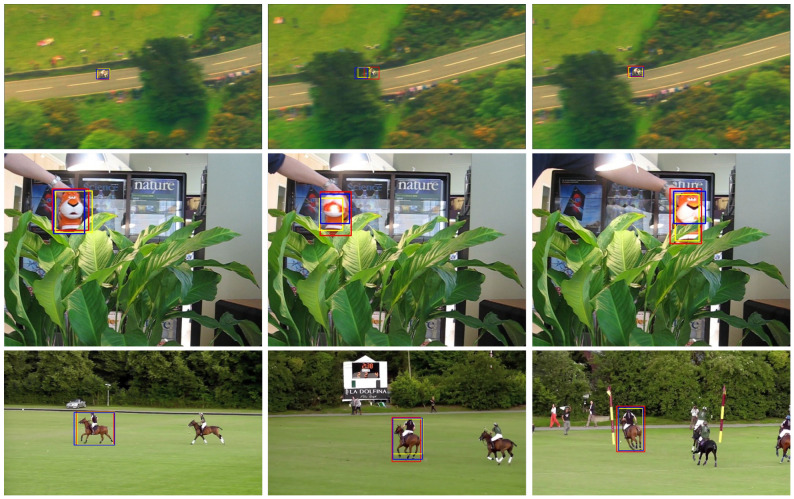
Visualizations of the tracking results of SiamGCN+CATUR compared to other trackers, SiamGCN [32] and SiamBAN [21], across three representative video sequences.

The visualization of tracking results is presented in Figure 7. The results in the first row of the figure show that, across consecutive frames of the same video sequence, SiamGCN + CATUR is the first to accurately re-localize the target immediately after occlusion, whereas SiamGCN and SiamBAN only re-capture the target in subsequent frames, indicating that CATUR accelerates the recovery from occlusion or target loss. The results in the second row further demonstrate that when the target is partially occluded, CATUR enhances SiamGCN’s ability to precisely localize the target, enabling the tracker to better adapt to complex scenarios. The images in the third row demonstrate that SiamGCN+CATUR achieves more precise localization of the target’s bounding box, exhibiting excellent bounding-box regression capabilities. The visualization results further validate the advantages of CATUR in improving tracking robustness and localization accuracy, particularly under challenging conditions such as occlusion, background blur, and scale variation, where it still achieves stable and precise target tracking.

### 4.4. Ablation Analysis

#### 4.4.1. Tracking Confidence Assessment

This section presents comparative experiments for various tracking confidence assessment methods, including the APCE-based method with dynamic thresholds proposed in this paper, maximum peak ($F_{\max}$), Peak-to-Sidelobe Ratio (PSR), and APCE with fixed thresholds. All experiments were conducted on the SiamGCN+CATUR model.

Experimental results in Table 5 show that the performance of methods using APCE, PSR, and $F_{max}$ as tracking confidence assessment metrics decreases in that order across these two datasets, which aligns with their respective levels of utilization of overall response map information. By measuring the overall distribution information of the response map, APCE is better able to suppress background interference, providing more accurate and stable tracking confidence, thereby enhancing the model’s tracking stability and accuracy. In contrast, PSR and $F_{max}$, which rely solely on local statistics, perform slightly worse on some metrics. This is because both methods employ a relatively simplistic approach to measuring response map peaks and fail to fully account for the influence of background noise. After applying the dynamic-threshold APCE calculation method proposed in this paper, the model’s performance is further improved compared to that of a fixed threshold.

Overall, APCE, as a tracking confidence metric, provides a relatively reliable basis for target-state judgment and template update strategies. When combined with the dynamic-threshold mechanism, its robustness and stability in complex scenarios are further enhanced.

#### 4.4.2. Adaptive Template Fusion

To evaluate the impact of template fusion strategies on tracking performance, this section designs a series of comparative experiments to compare traditional linear template fusion methods with adaptive template fusion methods, while also investigating the effect of different numbers of training stages on fusion performance. The experiments are conducted on the LaSOT and TrackingNet datasets, and the results are shown in Table 6. The experimental results indicate that when SiamGCN is used as the offline model, the adaptive template fusion method with three training stages can more fully integrate template information, significantly improving tracking accuracy and robustness, and yielding tracking performance that is markedly superior to that of the traditional linear update method. Furthermore, these results demonstrate that multi-stage training not only enhances the network’s ability to predict templates but also simulates, to some extent, the uncertainty introduced by localization errors during actual tracking.

#### 4.4.3. Module-Wise Ablation Experiments

This section presents ablation experiments for the two proposed modules: adaptive template fusion and target loss re-detection. Since the tracking confidence assessment module plays a critical role in assisting the model with template updates and target-loss re-detection, the ablation experiments for these two modules in this section are conducted with the tracking confidence assessment module included.

Detailed experimental results on the LaSOT and TrackingNet datasets are shown in Table 7. Here, TU denotes the use of tracking confidence assessment combined with adaptive template fusion, while RD denotes the use of tracking confidence assessment combined with target-loss re-detection. The simultaneous use of TU and RD constitutes the CATUR model described earlier. For comparison, this paper selects LightTrack as the reference model. On the one hand, LightTrack is a lightweight model and is comparable to the aforementioned SiamGCN in terms of design philosophy; on the other hand, LightTrack performs relatively well across a wide range of evaluations and serves as a highly valuable benchmark.

The experimental results show that both RD and TU effectively improve the overall performance of the tracking model, but they function differently. Specifically, the RD module enables the model to quickly re-detect the target after target loss, thereby effectively reducing the duration of tracking failures; the TU module primarily improves the model’s ability to adaptively update in response to target appearance variations, enabling the model to maintain high accuracy over long sequences. Consequently, RD yields more significant improvements in the AUC metric, while TU provides greater gains in PNorm and P. Taking the test results of SiamGCN on the two datasets as an example, the RD module improved AUC by 2.3% and 1.2%, respectively, but only increased precision P by 0.1% in both datasets; in contrast, the TU module increased P by 2.8% and 2.7%, respectively, while improving AUC by 1.7% and 2.9%, respectively.

The differences in the characteristics of the LaSOT and TrackingNet datasets also reflect this phenomenon. Since the LaSOT dataset contains more target-loss scenarios, the RD module performs more effectively on this dataset, significantly improving the AUC metric; whereas in the TrackingNet dataset, where video sequences are shorter and target loss is relatively rare, the TU module plays a more prominent role in enhancing tracking accuracy.

Furthermore, when the TU module and the RD module are used simultaneously, their complementary strengths enable the overall tracking performance to surpass that achieved when either module is applied independently. Models can quickly recover tracking after the target undergoes deformation, encounters temporary occlusion, or is lost, resulting in improved overall tracking robustness and precision.

Experiments on LightTrack, a lightweight SOTA model, also demonstrate that after integrating the TU and RD modules, the model outperforms the baseline version without these modules on most metrics, fully validating the effectiveness of combining tracking confidence assessment with adaptive template fusion and target-loss re-detection in enhancing tracking performance.

In summary, the ablation experiments above fully validate the practical value of the confidence-guided template update and re-detection network proposed in this paper when addressing complex tracking scenarios.

## 5. Conclusions

This paper proposes a confidence-guided adaptive template update and re-detection network (CATUR), which primarily consists of three components: a tracking confidence assessment module based on APCE, an adaptive template fusion network, and a global search strategy. Experiments demonstrate that the synergy among CATUR’s modules effectively improves the model’s tracking performance in dynamic scenarios.

However, the current experimental analysis is not yet exhaustive. Systematic sensitivity experiments should be further designed for the APCE-based tracking confidence module to better analyze the influence of parameters such as λ, $\gamma_{1}$, and $\gamma_{2}$. In addition, since NanoDet-plus is used as an external detector for re-detection, the use of a COCO-pretrained detector introduces additional dependencies. Although this paper focuses on confidence-guided template updates and re-detection triggering mechanisms rather than the design of the detector itself, results should be interpreted with caution when comparing this approach to trackers that do not rely on external detection models. Moreover, scenario-specific experiments, systematic time analysis, and failure case analysis should be further considered in future work to better evaluate the performance of the proposed method under different challenging conditions.

Future work will focus on evaluating CATUR’s frame rate (FPS), runtime overhead, and computational efficiency under various tracking conditions, and on improving the target verification strategy when the target undergoes long-term complete occlusion, severe deformation, or when multiple similar objects of the same category appear in the search frame. Our research currently focuses on a single model design, which may limit its generalizability. Therefore, future work will also focus on constructing a unified tracking framework to handle multiple tasks and broaden the range of applicable tracking scenarios.

## Figures and Tables

**Table 1:** Common Methods for Updating Templates.

Template Update Method	Core Idea	Mathematical Representation
Fixed template	The template remains unchanged	$T_{t}=T_{0}^{GT}$
Linear fusion	Weighted average of the current and historical templates	$T_{t}=\left(1-\alpha \right)T_{t-1}+\alpha T_{c}$
Nonlinear fusion	Learning fusion functions using neural networks	$T_{t}=f\left(T_{t-1},T_{c}\right)$
Template memory pool	Select the best template from the historical template pool	$T_{t}=\sum_{i=1}^{N}w_{i}T^{i}$

**Table 2:** Parameter settings for tracking confidence assessment.

Parameter Name	Parameter Value
*N*	5
λ	0.7
$\gamma_{1}$	0.5
$\gamma_{2}$	0.3

**Table 3:** Experimental Environment Configuration.

Parameter	Configuration
CPU	Intel(R) Core(TM) i7-12700H CPU @ 2.30 GHz
GPU	GeForce GTX 3090 Ti
Memory	90 GB
Storage	1 TB
Operating System	Linux
Programming Language	Python 3.7
Deep Learning Environment	PyTorch 2.1.0 + CUDA 12.2

**Table 4:** Detailed comparisons on LaSOT and TrackingNet. The best and second-best results are highlighted in red and blue fonts.

	LaSOT			TrackingNet		
	AUC	*P*_Norm_	P	AUC	*P*_Norm_	P
ECO [29]	32.4	33.8	30.1	55.4	61.8	49.2
MDNet [43]	41.3	48.1	37.4	60.6	70.5	56.5
C-RPN [19]	45.5	54.7	44.3	66.9	74.6	61.9
SiamMask [20]	46.7	55.2	46.9	72.5	77.8	66.4
SiamRPN++ [2]	49.6	56.9	49.1	73.3	80.0	69.4
ATOM [16]	51.5	57.6	50.5	70.3	77.1	64.8
CGACD [44]	51.8	62.6	51.1	71.1	80.0	69.3
LightTrack [45]	52.2	58.3	51.7	72.5	77.8	69.5
SiamGCN [32]	52.6	60.1	53.0	71.5	76.8	66.4
SiamGCN + CATUR	54.7	62.5	54.7	74.9	78.5	68.8

**Table 5:** Experimental results of different tracking confidence assessment methods. Bold values indicate the best result in each column.

Tracking Confidence Assessment Method	LaSOT			TrackingNet		
	AUC	*P*_Norm_	P	AUC	*P*_Norm_	P
$F_{\max}$	52.5	60.6	52.3	73.5	76.0	66.9
PSR	53.4	61.0	52.8	74.1	77.6	67.7
APCE	54.0	62.1	53.5	74.3	77.6	68.1
Dynamic-Threshold APCE	**54.7**	**62.5**	**54.7**	**74.9**	**78.5**	**68.8**

**Table 6:** Experimental Results of Different Template Fusion Methods. Bold values indicate the best result in each column.

Template Fusion Method	Number of Training Stages	LaSOT			TrackingNet		
		AUC	*P*_Norm_	P	AUC	*P*_Norm_	P
Linear Update	—	38.8	45.6	37.7	53.7	56.9	48.4
Adaptive Template Fusion	1	52.2	59.1	51.8	70.4	74.2	65.4
	2	53.6	61.1	53.1	71.2	73.8	66.0
	3	**54.7**	**62.5**	**54.7**	**74.9**	**78.5**	**68.8**
	4	52.5	60.0	52.5	70.8	73.7	66.2

**Table 7:** Ablation experiments on LightTrack [45] and SiamGCN. Bold values indicate the best result in each column.

	TU	RD	LaSOT			TrackingNet		
			AUC	*P*_Norm_	P	AUC	*P*_Norm_	P
LightTrack			52.2	58.3	51.7	72.5	77.8	69.5
	✓		52.9	60.0	52.9	74.0	78.9	70.1
		✓	53.1	59.4	52.9	72.8	78.1	69.6
	✓	✓	53.9	61.2	53.6	74.4	**79.7**	**70.3**
SiamGCN			52.6	60.1	53.0	71.5	76.8	66.4
	✓		53.5	61.9	54.5	73.6	78.2	68.2
		✓	53.8	61.0	53.4	72.3	77.3	66.9
	✓	✓	**54.7**	**62.5**	**54.7**	**74.9**	78.5	68.8

## Data Availability

Publicly available datasets were analyzed in this study. Further inquiries can be directed to the corresponding author. The code and additional implementation details are available from the corresponding author upon reasonable request.
